# Impacts of an egg intervention on nutrient adequacy among young Malawian children

**DOI:** 10.1111/mcn.13196

**Published:** 2021-05-11

**Authors:** Bess L. Caswell, Charles D. Arnold, Chessa K. Lutter, Lora L. Iannotti, Raphael Chipatala, Elizabeth Rochelle Werner, Kenneth M. Maleta, Christine P. Stewart

**Affiliations:** ^1^ Department of Nutrition University of California, Davis Davis California USA; ^2^ Food and Nutrition Division RTI International Research Triangle Park North Carolina USA; ^3^ Institute for Public Health Brown School at Washington University in Saint Louis St. Louis Missouri USA; ^4^ School of Public Health and Family Medicine University of Malawi College of Medicine Blantyre Malawi

**Keywords:** 24‐h recall, complementary feeding, eggs, infant, Malawi, nutrients, nutrition assessment

## Abstract

Eggs are a rich source of multiple nutrients that support child growth and development. Provision of eggs as a complementary food may improve dietary adequacy among young children at risk for undernutrition. Our objective was to test the impact of an egg intervention on the adequacy of total nutrient intakes and micronutrient density among 6‐ to 15‐month‐old Malawian children. Children 6 to 9 months old, living in Mangochi District, Malawi, were randomly assigned to the intervention group (*n* = 331) receiving an egg per day or a control group (*n* = 329) consuming their usual diet. Dietary intakes of macronutrients, vitamins and minerals were assessed using 24‐h recalls at baseline, 3‐month midline and 6‐month endline, with repeat recalls in a subsample. Usual nutrient intake and micronutrient density distributions were modelled to estimate group means and prevalence of inadequacy. Group differences at midline and endline were tested using unequal variance *t* tests with bootstrapped standard errors. The egg intervention resulted in higher intakes of fat and protein and lower intakes of carbohydrates. The egg group had lower prevalence of inadequacy for selenium, vitamin A, riboflavin, vitamin B_5_, vitamin B_12_ and choline. Micronutrient density inadequacy was lower in the egg group for vitamin A and choline at midline and endline, riboflavin at midline and vitamin B_5_ at endline. Inadequacy of nutrient intakes or density remained highly prevalent in both groups for multiple micronutrients. Though the egg intervention increased intakes of protein and several micronutrients, total intakes and micronutrient density of multiple micronutrients remained far below recommendations.

**Key messages**

- Eggs provide complete protein and several micronutrients critical for growth and development. This study analysed the impact of daily provision of eggs on nutrient intake adequacy among 6‐ to 15‐month‐old Malawian children.
- Though an egg complementary feeding intervention increased intakes of some nutrients essential for child growth and development, the diets of study participants remained highly inadequate in multiple micronutrients.
- To meet the micronutrient needs of young Malawian children, intensive programs to support dietary diversification and increase accessibility and use of multiple micronutrient powders or fortified foods may be required.

​

## 1 INTRODUCTION

Eggs are a rich source of multiple nutrients that support growth and development among young children, including protein with an optimal balance of amino acids to support requirements and vitamins and minerals such as vitamin A, vitamin B_12_, choline and selenium (Iannotti et al., 2014). Eggs provide these nutrients naturally and in a small, nutrient‐dense package. Thus, eggs may support child growth and development in populations characterized by high rates of child undernutrition and diets low in animal source foods or fortified foods (Iannotti et al., 2014).

It has been hypothesized that regular provision of eggs to children at risk of undernutrition may prevent stunting, which affects 22% of children under 5 years old globally and 33% in sub‐Saharan Africa (UNICEF, 2019). Eggs also provide nutrients that are essential for cognitive development (Iannotti et al., 2014). The Lulun Project, which provided eggs for daily consumption to 6‐ to 15‐month‐old children in rural Ecuador, resulted in marked improvements in linear growth and decreases in stunting prevalence (Iannotti et al., 2017). Children in the egg group had a mean length‐for‐age *Z*‐score 0.63 standard deviations (SDs) higher than children in the control group after 6 months of intervention, and stunting was reduced by 47% (Iannotti et al., 2017). In contrast, the Mazira Project, conducted among 6‐ to 15‐month‐old children in rural Malawi, resulted in improved head‐circumference‐for‐age but had no overall impact on growth in length or weight or on developmental outcomes (Prado et al., 2020; Stewart et al., 2019). The intervention had good adherence and an additive effect on complementary food intake equivalent to half an egg per day (Lutter et al., 2020; Stewart et al., 2019).

Given the limited impacts on growth and development of the Mazira Project in Malawi, strong results of the Lulun Project in Ecuador and critical role of dietary adequacy for healthy growth and development, it is important to examine nutrient intake changes associated with the egg intervention among Malawian children and identify nutrient intakes which may still fall below requirements. The background diet of children in Ecuador differs from that of children in Malawi (Iannotti et al., 2017; Lutter et al., 2020). Exploring the impacts of the egg intervention on nutrient intakes and nutrient intake adequacy may help explain why growth effects were observed in the Lulun Project but not in the Mazira Project, thereby providing insight into how programs to increase egg consumption may fit into child nutrition strategies for different populations. The present study is a secondary analysis of the Mazira Project examining total nutrient intakes from complementary feeding and estimated breast milk intake and micronutrient density of the complementary diet. Intakes of carbohydrates, fat, protein, calcium, iron, zinc, selenium, vitamin A, thiamin, riboflavin, niacin, vitamin B_5_, vitamin B_6_, folate, vitamin B_12_, vitamin C and choline were included in the analyses. Our objectives were to test the impact of the egg intervention on total nutrient intakes from complementary feeding and breast feeding, nutrient intake inadequacy, micronutrient density of the complementary diet and micronutrient density inadequacy among 6‐ to 15‐month‐old children in rural Malawi.

## 2 METHODS

### 2.1 Study context

The Mazira Project was a randomized controlled trial conducted in 2018–2019 to test the impact of daily egg consumption on growth and development among rural Malawian children age 6 to 15 months (clinicaltrials.gov: NCT03385252). The trial and primary outcomes have been previously described (Prado et al., 2020; Stewart et al., 2019). This study describes impacts on nutrient intakes as a secondary outcome.

Children living in the Lungwena and Malindi rural health centre catchment areas of Mangochi District, Malawi, were enrolled in the Mazira Project at 6 to 9 months of age. Age‐eligible children were recruited through rosters compiled by community health workers and community outreach. Primary caregivers of age‐eligible children were asked to report to the study clinic for enrolment and baseline assessments. Children were screened for the following exclusion criteria: history of egg allergy or any severe allergic reaction, inability to consume eggs safely in an on‐site test feeding, symptomatic malaria with positive rapid diagnostic test, severe anaemia (haemoglobin < 5 g/dl), moderate or severe acute malnutrition (mid‐upper arm circumference <12.5 cm), acute illness or injury warranting medical care, non‐singleton birth, or congenital condition or chronic illness that could impact growth, development or feeding. Caregivers participated in an information session about the study prior to completing the individual informed consent process and marking or signing a written consent form.

Baseline assessments included anthropometry, questionnaires on demographics, socio‐economic status and food security (Coates et al., 2007) and a 24‐h dietary recall. After completion of baseline assessments, children were individually, randomly assigned to the egg group or the control group (Stewart et al., 2019). Caregivers in the egg group received training on egg preparation methods and food safety in the first 2 weeks of study participation and a brief refresher training after 3 months. Throughout the 6‐month intervention, participants received twice‐weekly home visits for intervention delivery and monitoring of adherence and morbidity. During the two weekly visits, caregivers of children in the egg group received a total of 14 eggs per week: 7 eggs for the enrolled child and 7 eggs to share with other household members. Additionally, a 7‐day morbidity recall and 7‐day food frequency questionnaire focused on consumption of animal source foods were administered in both groups. To confirm adherence and safe feeding practices, staff conducted observations of the child being fed their daily egg during some of the home visits. A midline visit including anthropometry and a 24‐h dietary recall was completed in participants' homes. At 6 months after enrolment, an endline visit including anthropometry and a 24‐h dietary recall was conducted at the study clinic.

### 2.2 Dietary assessment

#### 2.2.1 The 24‐h dietary recall

Dietary data were collected by 24‐h recall with the primary caregiver at baseline, midline and endline for all participating children. Up to two repeat recalls at each time point were completed at participants' homes in a subsample of 100 children per group. Initial enrolment into the repeat recall subsample (14 March to 1 June 2018) included all children from 11 villages out of the 72 villages in the study area. The subsample villages were selected by stratified random sampling from traditional government administrative areas. Because enrolment was slower than anticipated, recruitment into the subsample was expanded to include all children enrolling in the trial in June 2018.

The 24‐h recall followed a multiple‐pass approach and was conducted using the Open Dietary Recall System (OpenDRS), an open‐access 24‐h recall form, deployed through SurveyCTO (Dobility, Inc., Cambridge, MA) on Android tablets (Caswell et al., 2019). OpenDRS can be adapted to different study contexts by loading lists of local foods and ingredients into the tablet form. For this study, the 24‐h recall form was populated with foods and ingredients reported in a previous study conducted in Mangochi District (Hemsworth et al., 2016). Interviewers completed 10 days of training on the 24‐h recall protocol using OpenDRS.

In the first pass of the 24‐h recall interview, the primary caregiver was asked to list all foods or drinks other than breast milk or water consumed by the child from waking the previous day until waking the day of the interview. Interviewers asked the caregiver to recall what the child had to eat or drink as they went about their daily activities, as well as whether any other caregivers might have given the child food or drinks. In the second pass, the caregiver was asked to describe each of the items listed in the first pass in detail. If an exact match to the food consumed was not in the pre‐set food list, the interviewer entered a description of the food. For any mixed dishes, the interviewer asked the caregiver to list the ingredients. In the third pass, the caregiver was asked to indicate the amount the child consumed of each food or drink. The interviewer asked the caregiver to estimate the amount of food or drink served to the child using uncooked rice, water or modelling dough, according to the texture of the food or drink being recalled. Local cups, dishes and spoons were used to aid estimation. The interviewer asked the caregiver to estimate the amount left over when the child finished eating, if any. The portion modelled by the caregiver was measured in millilitres using a graduated cylinder. In the final pass, the interviewer reviewed the reported recall, documenting any edits and recording any missed foods.

#### 2.2.2 Recipe data

Data on local recipes were collected through weighed cooking demonstrations. Women living in the study area, including mothers of children who had recently completed participation in the Mazira Project, were recruited to cook recipes at the study clinic using typical cookware and ingredients. Over 90 women, primarily from villages in the Lungwena catchment area, participated in two rounds recipe data collection (July 2018 and January 2019). One hundred twenty‐six recipes were selected to represent the mixed dishes most commonly reported in the 24‐h recalls conducted in the first 11 months out of the 12 months of study implementation, as well as the range of distinct ingredient combinations and preparation methods. Study staff weighed all ingredients and the final cooked recipes using food scales. Each recipe was prepared 5 to 20 times, with more replicates for the most common recipes. For less common recipes, the cooks were asked to portion out ingredient quantities they would use to prepare the recipes, and cooked weights were estimated using yield factors from similar recipes. Median ingredient proportions, expressed as percentage of cooked recipe weight, were used to create standard recipes. For uncommon mixed dishes, recipes were estimated based on ingredient proportions and yield factors from similar recipes. Staff measured the weight and volume of samples from cooked recipes and locally purchased foods and drinks to obtain density estimates.

#### 2.2.3 Calculation of observed nutrient intakes

To calculate observed nutrient intakes, we converted portion sizes recorded in millilitres to grams using local food density data or density values from the University of Minnesota Nutrition Data System for Research (NDSR) data tables, 2017 edition (Nutrition Coordinating Center—University of Minnesota, 2014). Mixed dishes were broken down into ingredient weights using local recipe data. Unmixed foods and ingredients from mixed dishes were linked to food composition data primarily from NDSR, supplemented by data on regional foods or from the USDA (FAO, 2017; Ferguson et al., 1989; Hotz et al., 2012; Joy et al., 2015; Kazembe et al., 2016; Nutrition Coordinating Center—University of Minnesota, 2014; Schakel, 2001; U.S.D.A.; Agricultural Research Service, 2019). Nutrient composition of a sample of locally purchased eggs was analysed (Eurofins Scientific Nutrition Analysis Center, Des Moines, Iowa) and included in the food composition table (Table S1). Energy and nutrient intakes from all foods and drinks reported in each recall were summed to estimate observed intakes from complementary feeding during the recall day.

Breast milk intake for each dietary recall day was estimated in kilocalories as the difference between the estimated energy requirement and energy intake calculated from the 24‐h recall (Hernandez et al., 2011; Morseth et al., 2018). Thus, breast milk intake could range from 0 kcal/day to a maximum of the estimated energy requirement for the child, if they had consumed no complementary foods that day. This approach was selected prior to analysis to allow for variation in breast milk intake, rather than assuming a fixed value for breast milk intake regardless of complementary food intake. The estimated energy requirement was calculated from formulas published by the Institute of Medicine (IOM), using the child's weight from the anthropometric assessment corresponding to 24‐h recall (IOM, 2006). Breast milk intake was converted from kilocalories to grams assuming an energy density of 0.65 kcal/g (World Health Organization [WHO], 1998). Published values for the nutrient contents of breast milk from IOM and the WHO were used to calculate nutrient intakes from breast milk (IOM, 2001, 2005; The Standing Committee on the Scientific Evaluation of Dietary Reference Intakes Panel on Folate, Other B Vitamins, and Choline, Subcommittee on Upper Reference Levels of Nutrients, & IOM Food and Nutrition Board, 1998; WHO, 1998; WHO et al., 2007).

#### 2.2.4 Protein quality adjustment

Crude protein intakes from complementary diet alone and from breastfeeding and complementary diet combined were adjusted to reflect the balance of amino acids relative to amino acid requirements using the Digestible Indispensable Amino Acid Score (DIAAS) (FAO, 2013). As recommended by the FAO, due to lack of published values for ileal amino acid digestibility, faecal protein digestibility factors were used in calculation of the DIAAS and adjusted amino acid and protein intakes (FAO, 2013; Food and Agriculture Organization, 1991; Food Policy and Food Science Service, 1981; Millward & Jackson, 2004). Protein from breast milk or infant formula was assumed to be fully digestible (Arsenault & Brown, 2017). The crude amino acid contents of each food were multiplied by its faecal protein digestibility. The digestibility‐adjusted amino acid contents of all foods and drinks and breast milk or for complementary foods and drinks only were then summed for each recall day and expressed as a ratio of available amino acid intake to crude protein intake. These ratios were divided by reference ratios published by the FAO for children age 6 months to 3 years. The lowest of these values became the DIAAS value and identified the limiting amino acid (FAO, 2013). The crude protein intake was multiplied by the DIAAS to estimate available protein intake.

#### 2.2.5 Statistical analysis

All statistical analyses were conducted in SAS 9.4 (SAS Institute, Cary, North Carolina). The statistical analysis plan was posted prior to starting analysis (https://osf.io/vfrg7/). *p* values less than 0.05 were considered significant unless otherwise stated. Descriptive statistics were used to summarize participant characteristics.

The National Cancer Institute (NCI) method for estimating usual nutrient intakes from 24‐h recall data with one recall per participant plus repeat recalls in a subsample was used for all dietary analyses (NCI Division of Cancer Prevention Biometry Research Group, n.d.). All recalls completed at the baseline, midline and endline visits were included in analyses of nutrient intakes. To reduce age or intervention effects on estimates of usual intakes, repeat recalls were excluded at baseline if they were completed after the participant's first intervention visit or more than 20 days after enrolment. Repeat recalls were similarly excluded if they were completed more than 10 days before or after the midline home visit or more than 20 days prior to the final clinic visit. For analyses of micronutrient density, recalls in which the caregiver reported that the child did not consume any complementary foods during the recall were also excluded (*n* = 14). Standard errors and 95% confidence intervals for each outcome measure were generated by analysing 200 bootstrap samples drawn by simple random sampling with replacement. At baseline, all participants were pooled in nutrient intake models because energy and animal source food intakes were previously found to be the same between the egg and control groups (Lutter et al., 2020; Stewart et al., 2019). At midline and endline, nutrient intakes, nutrient densities and prevalence of inadequacies in the egg and control groups were estimated separately by intervention group to estimate group means and account for potential differences in intake variance. Hypothesis testing of intervention effects was conducted using unequal variance *t* tests of differences in group mean usual intakes estimated by the usual nutrient intake distribution models and bootstrapped standard errors.

Distributions of usual nutrient intakes from complementary feeding and breast milk intake were modelled using the MIXTRAN (v.2.21) and DISTRIB (v.2) macros published by NCI (NCI Division of Cancer Prevention Biometry Research Group, n.d.). We included child age on day of recall interview and whether the recall period fell on a market day as covariates for all nutrients. Prior authors have recommended accounting for variation in intake on market days or found child dietary intakes to be lower on market days in low‐income settings (Caswell et al., 2018; Gibson & Ferguson, 2008). Based on exploratory analyses indicating that mean energy intake, and therefore total reported complementary food intake, was lower on market days in our study sample, we included market day as a fixed effect for all nutrients. Child sex and illness may impact nutrient intakes, but the presence, strength and direction of such effects may vary between nutrients. We therefore included child sex and caregiver report of child illness or unusual appetite during the recall period if these factors showed a bivariate association with nutrient intakes (*p* < 0.1). To estimate prevalence of inadequacy, we applied the nutrient intake requirement corresponding to the child's age on the day of the recall interview to pseudo‐individual intakes generated by the Monte Carlo simulation within the DISTRIB macro. This binary indicator of inadequacy was averaged across the simulated data to estimate prevalence of inadequacy. Estimated average requirements (EARs) or adequate intakes (AIs) for 6‐ to 12‐month‐old and 1‐ to 3‐year‐old children from the National Academies of Science, Engineering and Medicine (NASEM) were used as the cut‐offs for all nutrients except zinc and iron (NASEM, 2019). Given the dominance of maize porridge in participants' diets even in the presence of the egg intervention (Lutter et al., 2020) and the low contents of iron and zinc in eggs (Iannotti et al., 2014), we used EARs for zinc intake from plant‐based diets from the International Zinc Nutrition Consultative Group (IZiNCG) and EARs for iron intake from low bioavailability diets from the WHO (International Zinc Nutrition Consultative Group [IZiNCG], 2004; World Health Organization, 2004).

Nutrient density of complementary foods was defined as nutrient intake per 100 kcal (WHO, 1998). We applied the NCI method for estimating the distribution of a ratio of usual intakes, modelling the bivariate usual intake distribution of each micronutrient and energy using the NLMIXED_UNIVARIATE (v1.2), NLMIXED_BIVARIATE (v1.2) and DISTRIB_BIVARIATE (v1.1) macros and covariates as described above for the nutrient intake models (NCI Division of Cancer Prevention Biometry Research Group, n.d.; Freedman, Guenther, Dodd, et al., 2010; Freedman, Guenther, Krebs‐Smith, et al., 2010). Because some children had recall days on which they consumed no vitamin B_12_ or no vitamin C, we used the NCI procedure for incorporating both probability and amount of consumption in the ratio of an episodically consumed dietary component to a dietary component consumed daily (Freedman, Guenther, Dodd, et al., 2010). Prevalence of micronutrient density inadequacy was estimated by applying age‐specific desired micronutrient density cut‐points to the Monte Carlo simulation output from the DISTRIB_BIVARIATE macro.

Desired micronutrient density for complementary feeding was calculated using the below formula, adapted from Morseth et al. (2018).

$$\text{Desired micronutrient density}=\left[\frac{\mathrm{RDA},\mathrm{RNI}\;\text{or}\;\mathrm{AI}-\left(\text{concentration of nutrient in breast milk}\times \text{median breast milk intake}\right)}{\text{median energy intake from complementary food}}\right]\times 100$$

Desired micronutrient density was calculated for 6‐ to 8‐month‐old, 9‐ to 11‐month‐old and 12‐ to 23‐month‐old children using expected daily breast milk intake values from the WHO and median usual energy intake by age group from the 24‐h recall data (WHO, 1998). We used values for nutrient contents of breast milk as described above and recommended daily allowance (RDA), recommended nutrient intake (RNI) or AI values from NASEM, IZiNCG and WHO (IZiNCG, 2004; NASEM, 2019; WHO, 2004).

### 2.3 Ethical considerations

All protocols were reviewed and approved by the Research and Ethics Committee at the University of Malawi College of Medicine and the Institutional Review Board at the University of California, Davis.

## 3 RESULTS

The Mazira Project enrolled 660 children, with a mean age at baseline of 7.4 months (SD 1.2) (Table 1). The baseline prevalence of stunting was 14%. Almost all children were still breastfeeding at enrolment. For most child and household descriptors, the egg and control groups were similar. The control group had a higher percentage of households experiencing moderate or severe food insecurity (control group: 81%; egg group: 75%) and lower educational attainment among mothers (completion of primary school in the control group: 16%; egg group: 24%).

**TABLE 1:** Baseline characteristics of children enrolled in the Mazira Project, Malawi, 2018–2019, overall and by treatment group (*n* = 660)

Variable	All, mean ± SD or *n* (%)	Control group (*n* = 329), mean ± SD or *n* (%)	Egg group (*n* = 331), mean ± SD or *n* (%)
Age at enrolment (months)	7.4 ± 1.2	7.3 ± 1.2	7.4 ± 1.2
Female	319 (48%)	159 (48%)	160 (48%)
Stunted (< −2 LAZ)	90 (14%)	46 (14%)	44 (13%)
Underweight (< −2 WAZ)	52 (8%)	28 (9%)	24 (7%)
Wasted (< −2 WLZ)	7 (1%)	4 (1%)	3 (1%)
Consumption of animal source foods			
Fish	179 (27%)	74 (22%)	105 (32%)
Meat	12 (2%)	5 (2%)	7 (2%)
Eggs	27 (4%)	13 (4%)	14 (4%)
Dairy	56 (9%)	31 (9%)	25 (8%)
Breastfeeding	658 (100%)	329 (100%)	329 (100%)
Maternal age (years)	26.0 ± 6.7	26.1 ± 6.8	25.9 ± 6.7
Maternal education			
No formal education	119 (18%)	72 (22%)	47 (14%)
Incomplete primary school	409 (62%)	203 (62%)	206 (62%)
Completed primary school or more	132 (20%)	54 (16%)	78 (24%)
Number of children under 5	1.7 ± 0.8	1.7 ± 0.8	1.7 ± 0.8
Food insecure^a^	514 (78%)	267 (81%)	247 (75%)

^a^ Defined as moderate or severe food insecurity as assessed using the Household Food Insecurity Access Scale (Coates et al., 2007).

Abbreviation: SD, standard deviation.

**FIGURE 1:**
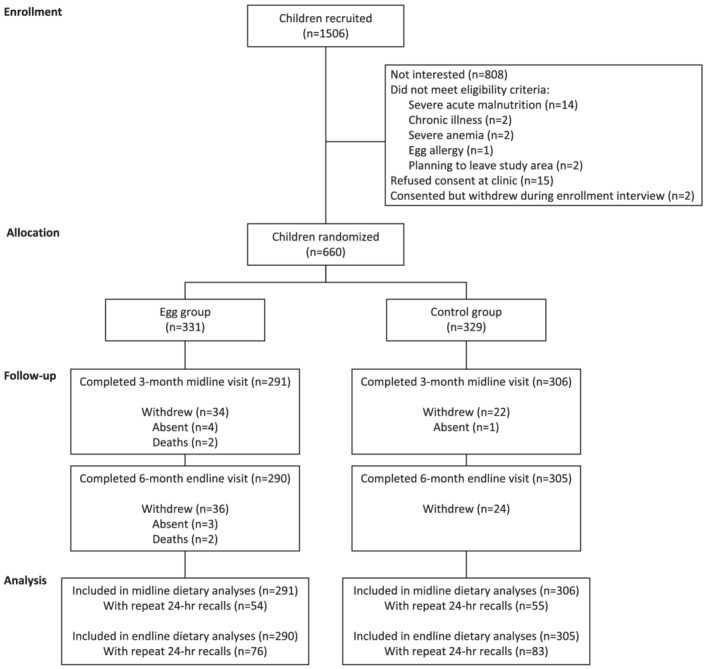
Participant flow diagram for dietary analyses of the Mazira Project, Mangochi District, Malawi, 2018–2019

Of 660 children enrolled in the Mazira Project, baseline dietary recalls were completed for 659 (Figure 1). Recalls were completed among 597 children at midline and 595 children at endline. At each time point, between 80 and 159 children completed one or more repeat recalls within the 21‐day inclusion window.

At baseline, protein intakes from complementary food and breast milk were adequate, even after adjusting for protein quality, but fat and carbohydrate intakes were inadequate among 60% and 81% of children, respectively (Table 2). Prevalence of inadequate intakes was greater than 90% for 10 out of fourteen micronutrients examined. Vitamin B_12_ was the only micronutrient assessed for which the prevalence of inadequacy was below 50%. The micronutrient density of complementary diets was very low relative to desired densities. Prevalence of micronutrient density inadequacy at baseline was 95% or greater for half of the examined micronutrients, and 80% or greater for another 3 out of 14 micronutrients.

**TABLE 2:** Baseline usual nutrient intakes, prevalence of intake inadequacy, micronutrient density and prevalence of micronutrient density inadequacy among 6‐ to 9‐month‐old children enrolled in the Mazira Project, Mangochi District, Malawi, 2018–2019 (*n* = 659)

Nutrient	EAR/AI^a^	Estimated usual intake per day, mean ± SE	Prevalence of nutrient intake inadequacy, % (95% CI)	Desired micronutrient density, intake per 100 kcal		Micronutrient density, mean ± SE	Prevalence of micronutrient density inadequacy, % (95% CI)
				Age 6–8 months	Age 9–11 months		
Carbohydrates (g)	95*	85 ± 0.7	81 (76, 85)	–	–	–	–
Fat (g)	30*	29 ± 0.3	60 (53, 66)	–	–	–	–
Crude protein (g)	1.12/kg	12 ± 0.1	0 (0, 0)	–	–	–	–
Availability‐adjusted protein (g)	0.95/kg	11 ± 0.1	0 (0, 0)	–	–	–	–
Calcium (mg)	260*	174 ± 3	94 (92, 97)	33	25	10.60 ± 1.50	95 (90, 100)
Iron (mg)	9.3	1.9 ± 0.05	100 (100, 100)	3.72	2.49	0.77 ± 0.07	100 (99, 100)
Zinc (mg)	4	1.58 ± 0.02	100 (100, 100)	2	1	0.48 ± 0.05	99 (98, 100)
Selenium (μg)	20*	19.7 ± 0.2	57 (52, 62)	3	2	4.01 ± 0.81	34 (26, 42)
Vitamin A (μg RAE)	500*	353 ± 5	94 (91, 98)	77	57	43.59 ± 2.93	84 (80, 88)
Thiamin (mg)	0.3*	0.259 ± 0.003	80 (76, 85)	0.07	0.05	0.05 ± 0.03	74 (48, 100)
Riboflavin (mg)	0.4*	0.287 ± 0.003	98 (96, 101)	0.07	0.05	0.04 ± 0.02	87 (61, 112)
Niacin (mg)	4*	1.88 ± 0.05	97 (95, 99)	1	1	0.69 ± 0.06	87 (78, 96)
Vitamin B_5_ (mg)	1.8*	1.22 ± 0.01	99 (98, 100)	0.28	0.21	0.11 ± 0.01	100 (94, 106)
Vitamin B_6_ (mg)	0.3*	0.201 ± 0.003	95 (93, 97)	0.1	0.07	0.06 ± 0.03	80 (51, 109)
Folate (μg DFE)	80*	72 ± 1	73 (64, 82)	10	8	11.26 ± 1.11	54 (44, 65)
Vitamin B_12_ (μg)	0.5*	0.63 ± 0.02	13 (−8, 34)	0^b^	0^b^	0.08 ± 0.12	0 (0, 0)
Vitamin C (mg)	50*	24.4 ± 0.4	100 (100, 100)	10	7	1.00 ± 0.08	100 (100, 100)
Choline (mg)	150*	102 ± 1	97 (95, 98)	19	15	6.79 ± 0.42	99 (97, 101)

*Note:* Usual nutrient intake and prevalence of nutrient intake inadequacy were estimated using the National Cancer Institute (NCI) method to control for measurement error and reflect combined intakes from complementary diet and breastfeeding. Standard errors and 95% confidence intervals calculated by bootstrap with *n* = 200 iterations. Micronutrient density of the complementary diet was calculated as nutrient intake per 100 kcal, using NCI method to model bivariate distributions of micronutrient and energy intakes, controlling for measurement error. Desired micronutrient density was calculated as the micronutrient density required to meet the recommended daily allowance or adequate intake of each nutrient, assuming published values for average breast milk intakes by age group and average energy intakes from complementary foods by age group from 24‐h recalls (World Health Organization [WHO], 1998). Prevalence of micronutrient density inadequacy was calculated as percent of participants with micronutrient density below the desired micronutrient density for their age.

Abbreviations: AI, adequate intake; CI, confidence interval; DFE, dietary folate equivalents; EAR, estimated average requirement; RAE, retinol activity equivalents; SE, standard error.

^a^ EAR or AI values are taken from the National Academies of Science, Engineering and Medicine for all nutrients other than iron and zinc (NASEM, 2019). The recommended nutrient intake for iron from low bioavailability diets is taken from the World Health Organization (WHO, 2004). The EAR for zinc from unrefined, plant‐based diets is taken from iZiNCG (IZiNCG, 2004). AIs are marked with an asterisk (*). The EAR for protein is expressed as gram protein per kilogram body weight. Inadequacy was determined using each child's body weight measured at the baseline assessment.

^b^ Desired micronutrient density for vitamin B_12_ is 0 for children age 6–8 and 9–11 months because breast milk intake is expected to provide sufficient vitamin B_12_ to meet requirements.

At midline and endline, children in the egg group had lower carbohydrate, higher fat and higher protein intakes than children in the control group (Table 3). Carbohydrate and fat intake inadequacy differed by group but was common among all participants. Protein intake inadequacy was 16% in the control group at midline and otherwise minimal. Crude and digestibility‐adjusted amino acid intakes were consistently higher in the egg group than in the control group (Table S2). Amino acid inadequacies were not observed, except for a 14% prevalence of lysine inadequacy in the control group at midline. Mean DIAAS values were higher in the egg group than the control group at both midline and endline (*p* < 0.0001). In the egg group, mean DIAAS was 94 at midline and 95 at endline. In the control group, mean DIAAS was 86 at midline and 88 at endline. In both groups and at all time points, the most frequently limiting amino acid was lysine.

**TABLE 3:** Usual macronutrient intakes and prevalence of inadequacy at 3‐month midline and 6‐month endline assessments, by treatment group, among children enrolled in the Mazira Project, Mangochi District, Malawi, 2018–2019

Nutrient	EAR/AI^a^		Egg group		Control group		*p* value^b^	
	Age 6–12 months	Age 1–3 years	Estimated usual intake per day, mean ± SE	Prevalence of inadequacy, % (95% CI)	Estimated usual intake per day, mean ± SE	Prevalence of inadequacy, % (95% CI)	Estimated usual intake	Prevalence of inadequacy
3‐month midline^c^								
Carbohydrates (g)	95*	100	92 ± 1.5	63 (57, 69)	98 ± 1.5	50 (42, 57)	0.003	0.004
Fat (g)	30*	ND	34 ± 0.6	33 (26, 39)	30 ± 0.5	52 (45, 60)	<0.0001	<0.0001
Crude protein (g)	1.12/kg	0.95/kg	17 ± 0.3	0 (0, 1)	15 ± 0.3	3 (0, 5)	<0.0001	0.11
Availability‐adjusted protein (g)	1.12/kg	0.95/kg	15 ± 0.3	2 (0, 4)	13 ± 0.3	16 (9, 23)	<0.0001	0.0003
6‐month endline^c^								
Carbohydrates (g)	95	100	98 ± 1.4	52 (42, 63)	105 ± 1.4	39 (30, 48)	0.0007	0.06
Fat (g)	30	ND	35 ± 0.6	–	31 ± 0.5	–	<0.0001	–
Crude protein (g)	1.12/kg	0.95/kg	19 ± 0.3	0 (0, 0)	17 ± 0.3	0 (0, 0)	<0.0001	0.54
Availability‐adjusted protein (g)	1.12/kg	0.95/kg	17 ± 0.3	0 (0,0)	15 ± 0.3	2 (0, 5)	<0.0001	0.10

*Note*: Usual nutrient intake and prevalence of nutrient intake inadequacy were estimated using the National Cancer Institute method to control for measurement error and reflect combined intakes from complementary diet and breastfeeding. Standard errors and 95% confidence intervals were calculated by bootstrap with *n* = 200 iterations.

Abbreviations: CI, confidence interval; SE, standard error.

^a^ Estimated average requirement (EAR) or adequate intake (AI) values are taken from the National Academies of Science, Engineering and Medicine or the WHO (NASEM, 2019; WHO et al., 2007). AIs are marked with an asterisk (*). Recommended intakes for fat among children age 1–3 years are not determined (ND) (NASEM, 2019).

^b^ Unequal variances *t* test for difference between groups using bootstrap standard errors (*n* = 200 bootstrap iterations).

^c^ Sample size at 3‐month midline included *n* = 291 children in the egg group and *n* = 306 children in the control group. Sample size at 6‐month endline included *n* = 290 children in the egg group and *n* = 305 children in the control group. Participants were 9 to 12 months old at midline and 12 to 15 months old at endline.

Intakes were higher, and prevalence of inadequacy was lower in the egg group than the control group for selenium, vitamin A, riboflavin, vitamin B_5_, vitamin B_12_ and choline at both midline and endline (Table 4). Folate intake was also higher and prevalence of inadequacy lower in the egg group at midline. Even with higher intakes of several nutrients in the egg group, prevalence of inadequacy remained high for many micronutrients. Prevalence of calcium, iron, niacin and vitamin B_5_ inadequacy was near or above 90% in both groups at midline and endline. Folate and choline inadequacies were also highly prevalent in both groups. The egg intervention was associated with a reduction in prevalence of inadequacy of vitamin A, riboflavin, vitamin B_5_, vitamin B_12_ and choline intakes at midline and riboflavin and vitamin B_12_ intakes at endline, though for all of these nutrients except vitamin B_12_, inadequacy remained above 40% in the egg group.

**TABLE 4:** Usual micronutrient intakes and prevalence of inadequacy at 3‐month midline and 6‐month endline assessments, by treatment group, among children enrolled in the Mazira Project, Mangochi District, Malawi, 2018–2019

	EAR/AI^a^		Egg group		Control group		*p* value^b^	
	Age 7–12 months	Age 1–3 years	Estimated usual intake per day, mean ± SE	Prevalence of inadequacy, % (95% CI)	Estimated usual intake per day, mean ± SE	Prevalence of inadequacy, % (95% CI)	Estimated usual intake	Prevalence of inadequacy
3‐month midline^c^								
Calcium (mg)	260*	500	174 ± 5	94 (91, 97)	176 ± 4	96 (92, 100)	0.77	0.54
Iron (mg)	9.3	5.8	2.6 ± 0.1	100 (99, 100)	2.5 ± 0.1	99 (99, 100)	0.24	0.54
Zinc (mg)	4	2	2.14 ± 0.07	80 (75, 85)	2.00 ± 0.05	83 (77, 88)	0.10	0.51
Selenium (μg)	20*	17	29 ± 0.5	3 (−1, 6)	23.6 ± 0.4	13 (2, 24)	<0.0001	0.09
Vitamin A (μg RAE)	500*	210	426 ± 9	59 (50, 69)	333 ± 8	71 (66, 75)	<0.0001	0.03
Thiamin (mg)	0.3*	0.4	0.281 ± 0.005	76 (70, 82)	0.287 ± 0.005	73 (67, 80)	0.38	0.59
Riboflavin (mg)	0.4*	0.4	0.403 ± 0.008	53 (45, 60)	0.303 ± 0.006	90 (84, 95)	<0.0001	<0.0001
Niacin (mg)	4*	5	2.45 ± 0.1	93 (88, 99)	2.62 ± 0.09	93 (89, 98)	0.21	0.99
Vitamin B_5_ (mg)	1.8*	2*	1.48 ± 0.03	89 (84, 95)	1.23 ± 0.02	99 (97, 100)	<0.0001	0.001
Vitamin B_6_ (mg)	0.3*	0.4	0.274 ± 0.006	77 (69, 85)	0.269 ± 0.006	80 (71, 89)	0.58	0.60
Folate (μg DFE)	80*	120	85 ± 2	61 (53, 69)	79 ± 2	70 (60, 80)	0.036	0.17
Vitamin B_12_ (μg)	0.5*	0.7	1.18 ± 0.05	1 (−2, 4)	0.94 ± 0.05	16 (3, 30)	0.001	0.04
Vitamin C (mg)	50*	13	22.8 ± 0.7	72 (66, 77)	24.1 ± 0.7	73 (69, 78)	0.19	0.64
Choline (mg)	150*	200*	138 ± 3	78 (70, 86)	96 ± 2	98 (97, 100)	<0.0001	<0.0001
6‐month endline^c^								
Calcium (mg)	260*	500	209 ± 6	99 (98, 99)	202 ± 5	99 (98, 100)	0.39	0.95
Iron (mg)	9.3	5.8	3 ± 0.1	98 (97, 100)	2.8 ± 0.1	100 (99, 101)	0.16	0.09
Zinc (mg)	4	2	2.75 ± 0.07	20 (11, 28)	2.59 ± 0.08	26 (15, 37)	0.15	0.40
Selenium (μg)	20*	17	31.2 ± 0.5	0 (0, 0)	26.3 ± 0.4	1 (−1, 2)	<0.0001	0.39
Vitamin A (μg RAE)	500*	210	408 ± 9	9 (5, 12)	335 ± 9	15 (7, 23)	<0.0001	0.13
Thiamin (mg)	0.3*	0.4	0.306 ± 0.005	87 (83, 91)	0.315 ± 0.005	90 (85, 95)	0.18	0.39
Riboflavin (mg)	0.4*	0.4	0.416 ± 0.007	43 (35, 52)	0.327 ± 0.005	89 (82, 96)	<0.0001	<0.0001
Niacin (mg)	4*	5	3.15 ± 0.09	92 (87, 96)	3.21 ± 0.1	92 (87, 97)	0.67	0.87
Vitamin B_5_ (mg)	1.8*	2*	1.47 ± 0.02	95 (92, 99)	1.26 ± 0.02	99 (98, 100)	<0.0001	0.024
Vitamin B_6_ (mg)	0.3*	0.4	0.317 ± 0.007	81 (76, 85)	0.307 ± 0.006	87 (79, 94)	0.25	0.17
Folate (μg DFE)	80*	120	84 ± 2	92 (87, 98)	84 ± 2	89 (84, 93)	0.91	0.28
Vitamin B_12_ (μg)	0.5*	0.7	1.69 ± 0.07	0 (−1, 2)	1.35 ± 0.07	14 (1, 27)	0.001	0.037
Vitamin C (mg)	50*	13	22.1 ± 0.7	26 (21, 32)	23.2 ± 0.8	23 (18, 29)	0.31	0.47
Choline (mg)	150*	200*	134 ± 3	97 (93, 100)	99 ± 2	100 (99, 100)	<0.0001	0.10

*Note:* Usual nutrient intake and prevalence of nutrient intake inadequacy were estimated using the National Cancer Institute method to control for measurement error and reflect combined intakes from complementary diet and breastfeeding. Standard errors and 95% confidence intervals calculated by bootstrap with n = 200 iterations.

Abbreviations: AI, adequate intake; DFE, dietary folate equivalents; EAR, estimated average requirement; RAE, retinol activity equivalents; SE, standard error.

^a^ EAR or AI values are taken from the National Academies of Science, Engineering and Medicine for all nutrients other than iron and zinc (NASEM, 2019). RNIs for iron from low bioavailability diets are taken from the World Health Organization (WHO, 2004). EARs for zinc from unrefined, plant‐based diets are taken from iZiNCG (IZiNCG, 2004). AIs are marked with an asterisk (*).

^b^ Unequal variances *t* test for difference between groups using bootstrap standard errors (*n* = 200 bootstrap iterations).

^c^ Sample size at 3‐month midline included n = 291 children in the egg group and n = 306 children in the control group. Sample size at 6‐month endline included n = 290 children in the egg group and n = 305 children in the control group. Participants were 9 to 12 months old at midline and 12 to 15 months old at endline.

Micronutrient density of the complementary diet was higher and micronutrient density inadequacy lower in the egg group than in the control group for vitamin A and choline at midline and endline, riboflavin at midline and vitamin B_5_ at endline. However, micronutrient density inadequacy remained above 40% in the egg group for vitamin A, riboflavin and choline at midline and near or above 90% for vitamin B_5_ and choline at endline. Prevalence of micronutrient density inadequacy was very high in both groups for the majority of micronutrients assessed. Prevalence of micronutrient density inadequacy was low only for selenium at both time points and vitamin A at endline (Table 5).

**TABLE 5:** Usual micronutrient density and prevalence of micronutrient density inadequacy at 3‐month midline and 6‐month endline assessments, by treatment group, among children enrolled in the Mazira Project, Mangochi District, Malawi, 2018–2019

	Desired micronutrient density		Egg group (*n* = 301)		Control group (*n* = 296)		*p* value^a^	
	Age 9–11 months	Age 12–23 months	Micronutrient density, mean ± SE	Prevalence of inadequacy, % (95% CI)	Micronutrient density, mean ± SE	Prevalence of inadequacy, % (95% CI)	Micronutrient density	Prevalence of inadequacy
3‐month midline^b^								
Calcium (mg)	25	121	13.76 ± 0.98	94 (90, 99)	14.48 ± 1.04	94 (90, 98)	0.62	0.95
Iron (mg)	2.49	1.24	0.60 ± 0.07	100 (97, 103)	0.65 ± 0.05	98 (95, 100)	0.49	0.28
Zinc (mg)	1	1	0.53 ± 0.07	92 (84, 100)	0.46 ± 0.04	95 (92, 99)	0.40	0.48
Selenium (μg)	2	2	5.61 ± 0.93	0 (0, 1)	4.24 ± 0.35	3 (−2, 8)	0.17	0.25
Vitamin A (μg RAE)	55	7	116.21 ± 11.66	48 (39, 56)	27.37 ± 2.58	71 (65, 77)	<0.0001	<0.0001
Thiamin (mg)	0.05	0.09	0.06 ± 0.01	57 (43, 72)	0.05 ± 0.01	68 (51, 85)	0.36	0.35
Riboflavin (mg)	0.05	0.07	0.07 ± 0.01	43 (30, 55)	0.04 ± 0.003	81 (71, 91)	0.0005	<0.0001
Niacin (mg)	1	1	0.59 ± 0.11	93 (77, 109)	0.64 ± 0.05	88 (78, 99)	0.72	0.66
Vitamin B_5_ (mg)	0.21	0	0.19 ± 0.03	78 (65, 90)	0.15 ± 0.02	88 (80, 96)	0.22	0.17
Vitamin B_6_ (mg)	0.07	0.1	0.08 ± 0.02	60 (41, 78)	0.06 ± 0.02	72 (47, 96)	0.47	0.44
Folate (μg DFE)	23	23	12.41 ± 0.95	39 (31, 48)	11.55 ± 1.13	48 (38, 58)	0.56	0.20
Vitamin B_12_ (μg)	0	0.08	0.19 ± 0.33	73 (63,82)	0.17 ± 0.03	82 (75, 89)	0.94	0.12
Vitamin C (mg)	7	0	1.62 ± 1.21	99 (95, 103)	2.44 ± 42.04	96 (89, 103)	0.98	0.5
Choline (mg)	13	27	16.70 ± 1.25	58 (48, 68)	6.5 ± 0.87	98 (96, 100)	<0.0001	<0.0001
6‐month endline^b^								
Calcium (mg)	25	121	21.30 ± 3.84	98 (95, 100)	19.18 ± 1.23	98 (97, 100)	0.60	0.56
Iron (mg)	2.49	1.24	0.73 ± 0.1	90 (83, 96)	0.69 ± 0.06	92 (84, 99)	0.69	0.72
Zinc (mg)	1	1	0.47 ± 0.05	99 (91, 106)	0.61 ± 0.06	87 (77, 97)	0.07	0.06
Selenium (μg)	2	2	4.96 ± 5.42	0 (0, 1)	4.05 ± 0.48	1 (−1, 4)	0.87	0.53
Vitamin A (μg RAE)	55	7	49.27 ± 9.6	6 (4, 9)	28.34 ± 1.89	14 (8, 21)	0.033	0.022
Thiamin (mg)	0.05	0.09	0.04 ± 0.01	88 (74, 103)	0.06 ± 0.01	75 (57, 93)	0.23	0.24
Riboflavin (mg)	0.05	0.07	0.04 ± 0.01	78 (59, 98)	0.04 ± 0.003	99 (90, 108)	0.85	0.06
Niacin (mg)	1	1	0.62 ± 0.05	94 (86, 103)	0.67 ± 0.07	93 (78, 108)	0.56	0.94
Vitamin B_5_ (mg)	0.21	0	0.18 ± 0.02	98 (97, 99)	0.13 ± 0.01	99 (98, 100)	0.029	0.034
Vitamin B_6_ (mg)	0.07	0.1	0.04 ± 0.02	93 (74, 111)	0.07 ± 0.02	76 (57, 96)	0.26	0.24
Folate (μg DFE)	23	23	11.52 ± 0.96	91 (87, 95)	11.28 ± 0.54	90 (87, 94)	0.83	0.88
Vitamin B_12_ (μg)	0	0.08	0.25 ± 0.55	8 (−23, 38)	0.18 ± 0.02	23 (8, 38)	0.90	0.39
Vitamin C (mg)	7	0	1.33 ± 1.39	100 (99, 101)	1.48 ± 0.2	100 (100, 100)	0.92	0.95
Choline (mg)	13	27	16.72 ± 4.43	88 (83, 94)	7.75 ± 0.43	99 (98, 101)	0.045	0.0001

*Note:* Micronutrient density of the complementary diet was calculated as nutrient intake per 100 kcal, using National Cancer Institute (NCI) method to model bivariate distributions of micronutrient and energy intakes, controlling for measurement error. Desired micronutrient density was calculated as the micronutrient density required to meet the recommended daily allowance or adequate intake of each nutrient, assuming published values for average breast milk intakes by age group and average energy intakes from complementary foods by age group from 24‐h recalls (WHO, 1998). Desired micronutrient density is set to 0 if average breast milk intakes provide nutrient intakes at or above the recommended nutrient intake level. Prevalence of micronutrient density inadequacy was calculated as percent of participants with micronutrient density below the desired micronutrient density for their age.

Abbreviations: DFE, dietary folate equivalents; RAE, retinol activity equivalents; SE, standard error.

^a^ Unequal variances *t* test for difference between groups using bootstrap standard errors (*n* = 200 bootstrap samples).

^b^ Sample size at 3‐month midline included *n* = 291 children in the egg group and *n* = 306 children in the control group. Sample size at 6‐month endline included *n* = 290 children in the egg group and *n* = 305 children in the control group. Participants were 9 to 12 months old at midline and 12 to 15 months old at endline.

## 4 DISCUSSION

We assessed the impact of the Mazira Project egg intervention on nutrient intakes, density and adequacy among rural Malawian children age 6 to 15 months. We found that the intervention increased total nutrient intakes and micronutrient density of the complementary diet for a number of nutrients found in high levels in eggs. Total intakes of fat, protein, selenium, vitamin A, riboflavin, vitamin B_12_ and choline were higher in the egg group than the control group. This resulted in reduced prevalence of inadequacy for most of these nutrients. Nutrient density adequacy was improved for several micronutrients, particularly vitamin A and choline. Prevalence of inadequate nutrient intake or density remained above 80% for calcium, iron, zinc, thiamin, niacin, vitamin B_5_, vitamin B_6_, folate and choline at endline. Deficiencies of these nutrients may cause anaemia or result in impaired growth, immune function or cognitive function and development (Mann & Truswell, 2007).

Intakes of available protein were largely adequate, and carbohydrate intakes were low. Fat intakes were also low in both groups, despite increases in fat intake from eggs and cooking oil among children in the intervention group (Lutter et al., 2020). Energy intakes from complementary foods were generally adequate (Lutter et al., 2020), so the estimates of adequate protein and low carbohydrates and fat may be due to the fact that the protein requirements are expressed on a per kilogram bodyweight basis while the carbohydrate and fat requirements assume an average body size (The Panel on Macronutrients et al., 2005). Mazira Project participants had a low mean weight for age relative to the WHO growth standards (Stewart et al., 2019).

Because previous studies have reported that animal protein intake was related to growth among 12‐ to 36‐month‐old Malawian children (Ghosh, 2016; Kaimila et al., 2019), we explored the contribution of eggs, a source of complete protein (Iannotti et al., 2014), to availability‐adjusted protein and amino acid intakes. Though the DIAAS values indicated that the egg intervention improved the balance of amino acids in the diet relative to amino acid requirements, crude protein intakes and protein quality were largely sufficient to meet protein and amino acid requirements in both treatment groups.

The estimates of nutrient intakes and micronutrient densities observed in both the egg and control groups of the Mazira Project in Malawi are largely similar to those recently reported among 6‐ to 15‐month‐old children elsewhere in sub‐Saharan Africa, with differences in specific micronutrients attributable to intakes of key foods such as fish in Malawi, fortified staples and infant foods in South Africa or milk in Ethiopia (Faber et al., 2016; Mengistu et al., 2017; Swanepoel et al., 2019). A survey conducted in 1998 in a district adjacent to the Mazira Project study area reported micronutrient densities largely similar to those we observed, indicating little change in micronutrient adequacy of complementary diets among young Malawian children over the past two decades (Hotz & Gibson, 2001). We have previously described the background diet of children in this study as consisting primarily of maize porridge, with limited frequency and small portions of micronutrient‐rich side dishes such as leafy green vegetables, beans or meat (Lutter et al., 2020).

Like the Mazira Project, several previous complementary feeding trials have found that nutrient intakes were increased in the supplemented group (Adu‐Afarwuah et al., 2007; Campbell et al., 2018; Flax et al., 2015; Hemsworth et al., 2016; Ickes et al., 2015; Thakwalakwa et al., 2015). In a trial of small‐quantity lipid‐based nutrient supplements (LNS) conducted in the same district of Malawi as the Mazira Project, children receiving 10‐ to 40‐g LNS per day had higher macronutrient intakes than children in a control group (Hemsworth et al., 2016). Another complementary feeding trial in the same area showed that both fortified porridge mix and LNS increased calcium, iron, zinc and vitamin C intakes (Thakwalakwa et al., 2015). Other complementary feeding trials of LNS or fortified porridge in Bangladesh, Honduras and Ghana have similarly demonstrated increased nutrient intakes (Adu‐Afarwuah et al., 2007; Campbell et al., 2018; Flax et al., 2015). Like these studies, this analysis explores the additive effect of a community‐based child feeding intervention, accounting for compliance and the possibility of displacement of other foods. A critical difference between these analyses and the present study is that the other trials examined processed, fortified complementary feeding supplements designed to provide required amounts of multiple micronutrients. In contrast, eggs provide micronutrients and complete protein with minimal processing, and in many settings, they can be produced locally (Iannotti et al., 2014).

The Mazira Project was designed to be comparable to the Lulun Project in Ecuador, yet the Lulun Project showed substantial impacts on growth and rate of stunting, while the Mazira Project did not (Iannotti et al., 2017; Stewart et al., 2019). The Lulun Project reported food frequencies rather than nutrient intakes, so we are unable to make a direct comparison to our results (Iannotti et al., 2017). However, a separate study conducted in 2009 reported intakes of protein, calcium, iron, zinc, vitamin A and vitamin C in two contiguous provinces to the site of the Lulun Project in the rural Ecuadorian highlands (Roche et al., 2017). Compared to children of similar ages in that study, Mazira Project participants in both groups at baseline and the control group at midline and endline had lower nutrient density for vitamin A, vitamin C and calcium, similar zinc density and higher iron density of the complementary diet. Total intakes of protein, vitamin C, iron and zinc among Malawian children were lower than intakes of these nutrients from complementary foods alone among the Ecuadorian children. If nutrient intakes among Lulun Project participants were similar to those of children in neighbouring provinces, there may have been smaller gaps between intake and requirement among children in Ecuador than those we observed in Malawi.

The possibility that the increased nutrient intakes from the egg intervention were insufficient to address growth‐limiting nutrient deficits is strengthened by findings from a trial of LNS that was previously conducted in the same district as the Mazira Project. The ILINS‐DOSE project found that children supplemented with 10–40 g of LNS did not have improved growth compared to children in a control group, despite the supplementation having an additive effect on energy and macronutrient intakes (Hemsworth et al., 2016; Maleta et al., 2015). The authors hypothesized that total delivery and adherence in the LNS groups may have been too low to provide the RDAs of micronutrients with which the LNS was fortified (Maleta et al., 2015).

We have previously proposed an alternative explanation for the differing results of the Lulun Project and Mazira Project that the frequent consumption of fish in this population may have limited the potential for benefit from a complementary feeding intervention (Stewart et al., 2019). Though consumption of mixed dishes containing fish was reported for 63% of all participants at endline, portion sizes were small and caregivers frequently reported that the child ate only the sauce from the mixed dish. The consumption of fish may help explain our finding that vitamin B_12_ intakes were adequate for 98% of children in the egg group and 86% of children in the control group at endline, yet the overall diet remains insufficient to meet requirements for most other micronutrients.

Eggs can provide some, but not all, of the micronutrients with high rates of inadequacy among young Malawian children. Several nutrients not found in high quantities in eggs, such as calcium, iron, several B vitamins and vitamin C, were deeply lacking in the diets of Mazira Project participants. For some nutrients found in eggs, background intakes were so low (e.g., choline) or so high (e.g., vitamin B_12_) that the egg intervention had modest impacts on the prevalence of inadequacy despite increasing intakes. To meet the micronutrient needs of this population, much more intensive programs supporting dietary diversification and delivering multiple micronutrient powders or fortified foods may be required, along with programs to address other constraints on growth such as infectious disease (Millward, 2017).

We used 24‐h dietary recalls at baseline, midline and endline with repeat recalls in a subsample of participants to conduct a detailed analysis of nutrient intakes and micronutrient density among participants in the Mazira Project. The collection of detailed dietary intake data with repeat observations throughout the course of the intervention is a key strength of this study. An additional strength is our application of tools from NCI to model usual nutrient intakes and estimate prevalence of intake inadequacy after controlling for measurement error associated with day‐to‐day intake variation (NCI Division of Cancer Prevention Biometry Research Group, n.d.). We applied methods described by NCI for estimating ratios of usual intake of two nutrients to nutrient density and nutrient density adequacy of the complementary diet (Freedman, Guenther, Dodd, et al., 2010). This approach was originally demonstrated for the calculation of percent energy from macronutrients or adherence to food‐based dietary guidelines (Freedman, Guenther, Dodd, et al., 2010; Freedman, Guenther, Krebs‐Smith, et al., 2010). To our knowledge, our use of this method to estimate usual nutrient density and prevalence of nutrient density inadequacy is novel. We have also used the DIAAS method to adjust protein intakes for digestibility and availability relative to the required balance of amino acids (FAO, 2013).

Our study is subject to the same limitations of all studies that rely on 24‐h dietary recall data, including omitted or added foods, errors in estimation of portion sizes and missing or inaccurate food composition data (Gibson, 2005). The study had a 10% loss to follow‐up at endline, but the numbers and baseline characteristics of children who were lost to follow‐up did not differ by treatment group, so we did not control for missing data in our analyses (Stewart et al., 2019). A further limitation is the extent to which our results, especially some micronutrient intakes, are generalizable to other populations. The Mazira Project was conducted in a small study area with the uncommon food system feature of high fish accessibility, due to the proximity to Lake Malawi. Children in other parts of Malawi or other low‐income countries may have lower intakes of micronutrients found in highest amounts in animal source foods, such as vitamin B_12_ (Dror & Allen, 2011).

A limitation of our analytic approach is that using bootstrapping to generate standard errors for prevalence of inadequacy yields *p* values and confidence intervals that must be interpreted cautiously for very high or very low prevalence estimates. Where this method has yielded negative values for the lower confidence interval bound, we have retained it to acknowledge the imprecision in the estimate. A further potential methodological limitation is that we have not applied a correction for multiple hypothesis testing to the *p* values calculated from the bootstrap standard errors. However, the statistical tests are not independent due to the nature of the correlation of the nutrients within the food provided, and our key findings remain well below the *p* value threshold we would have obtained using a conservative Bonferroni correction based on analysis of 18 nutrients at each time point. We have interpreted our findings in consideration of the nutrient composition of eggs and the nutritional or public health significance of observed differences. Given these considerations, our conclusions are unlikely to be substantially affected by these methodological limitations.

Our estimates of nutrient intakes and prevalence of nutrient intake inadequacy should be understood in the context of assumptions we made about nutrient intakes from breast milk. First, we used estimates of the nutrient contents of breast milk from the WHO and IOM (IOM Food and Nutrition Board, 2001; The Panel on Macronutrients et al., 2005; The Standing Committee on the Scientific Evaluation of Dietary Reference Intakes Panel on Folate, Other B Vitamins, and Choline et al., 1998; WHO, 1998). These estimates are averages over studies conducted in other countries and may not be accurate for our study population. Second, to estimate breast milk intake, we relied on a previously published method of assuming that each child's energy intake was equivalent to their estimated energy requirement (Hernandez et al., 2011; Morseth et al., 2018). Given the very low rates of wasting and no observations of overweight among study participants (Table 1), it is reasonable to assume that energy intake is, on average, equal to requirements. However, this assumption may result in underestimation of breast milk intake. The average estimated breast milk intake in our analysis was lower than the WHO estimated intakes by age group (WHO, 1998). Third, we recently reported that children in the egg group consumed about 30 kcal more per day from complementary feeding than children in the control group, though this result was not statistically significant (Lutter et al., 2020). Therefore, our assumption about breast milk intake results in a more conservative estimate of between‐group differences in nutrient intake; if there is no displacement of breast milk intake due to the intervention, total nutrient intakes in the egg group would be even higher relative to those in the control group than what we estimate. Finally, our findings on the adequacy of total nutrient intakes parallel our findings on the adequacy of micronutrient density, which does not incorporate assumed breast milk intake. Therefore, the conclusion that the egg intervention increased nutrient intakes yet micronutrient intakes remain well below the requirements is unlikely to be affected by our assumption on breast milk intake.

In this dietary assessment of the Mazira Project complementary feeding trial, we found that provision of eggs for daily consumption to rural Malawian children age 6 to 15 months improved micronutrient intakes and intake adequacy for nutrients of which eggs are a rich source, such as vitamin A, riboflavin, vitamin B_5_, vitamin B_12_, selenium and choline (Iannotti et al., 2014). The egg intervention also increased protein and amino acid intakes. However, total micronutrient intakes and micronutrient density of the complementary diet remained critically low for many micronutrients, potentially limiting child growth and development (WHO, 1998). Provision of multiple micronutrient powder, fortified food or additional nutrient‐dense complementary foods may be needed to fill the nutrient gaps.

## CONFLICTS OF INTEREST

The authors declare that they have no conflicts of interest.

## CONTRIBUTIONS

The Mazira Project randomized controlled trial was designed by CPS, CKL, KMM, LLI and ELP and conducted by CPS, KMM, BLC and CDA. Dietary data collection was designed and managed by BLC. Recipe data collection was conducted by ERW and BLC. Data analysis was performed by BLC and CDA. BLC wrote the manuscript. All authors provided critical revisions and approved the final version.

## Supporting information


**Table S1.** Nutrient composition of eggs purchased in Mangochi District, Malawi, 2018 and nutrient composition of eggs listed in FoodData Central^†^
Table S2. Usual crude and digestibility‐adjusted amino acid intakes at 3‐month midline and 6‐month endline assessments, by treatment group, among children enrolled in the Mazira Project, Mangochi District, Malawi, 2018–2019^†^

## Data Availability

The data that support the findings of this study are openly available on the Open Science Framework (at https://osf.io/vfrg7/or http://doi.org/10.17605/OSF.IO/VFRG7), title “The Mazira Project” (Arnold et al., 2018).
