# Supplementary material for: Impacts of an egg intervention on nutrient adequacy among young Malawian children
Source: Matern Child Nutr. 2021 May 11;17(3):e13196. doi: 10.1111/mcn.13196 (PMC8189245; doi:10.1111/mcn.13196)
Supplement: Supplementary file 1 — Table S1. Nutrient composition of eggs purchased in Mangochi District, Malawi, 2018 and nutrient composition of eggs listed in FoodData Central† Table S2. Usual crude and digestibility‐adjusted amino acid intakes at 3‐month midline and 6‐month endline assessments, by treatment group, among children enrolled in the Mazira Project, Mangochi District, Malawi, 2018–2019† [file MCN-17-e13196-s001.docx]

Supplementary Table 1. Nutrient composition of eggs purchased in Mangochi District, Malawi, 2018 and nutrient composition of eggs listed in FoodData Central^†^

|  | Eggs purchased in Mangochi, Malawi | FoodData Central |
| --- | --- | --- |
| Energy (kcal/100g) | 143 | 155 |
| Carbohydrates (g/100g) | <LOD^‡^ | 1.12 |
| Fats (g/100g) | 10.27 | 10.61 |
| Protein (g/100g) | 12.5 | 12.6 |
| Calcium (mg/100g) | 47 | 50 |
| Iron (mg/100g) | 1.7 | 1.2 |
| Zinc (mg/100g) | 1.1 | 1.1 |
| Selenium (μg/100g) | 21.0 | 30.8 |
| Vitamin A (μg RAE/100g)^‡^ | 150 | 149 |
| Folate (μg DFE/100g)^‡^ | 63.5 | 44 |
| Vitamin B_12_ (μg/100g) | 1.61 | 1.11 |
| Choline (mg/100g) | 238 | 294 |

^†^Values are reported content per 100g of whole, boiled egg. Eggs from Malawi were purchased from Mangochi town in July 2018, hard-boiled and shipped on ice. Analysis of the single, pooled sample of 11 eggs was done by Eurofins Scientific Nutrition Analysis Center (Des Moines, IA). The FoodData Central record 173424 for Egg, whole, cooked, hard-boiled is provided here for reference (U.S. Department of Agriculture; Agricultural Research Service, 2019); it was not used in analyses.

^‡^LOD, limit of detection; RAE, Retinol Activity Equivalents; DFE, Dietary Folate Equivalents.

Supplementary Table 2. Usual crude and digestibility-adjusted amino acid intakes at 3-month midline and 6-month endline assessments, by treatment group, among children enrolled in the Mazira Project, Mangochi District, Malawi, 2018-2019^†^

|  | Egg group | | | Control group | | | p-value^‡^ | | |
| --- | --- | --- | --- | --- | --- | --- | --- | --- | --- |
|  | Usual crude intake,  mean ± SE | Usual adjusted intake,  mean ± SE | Prevalence of inadequacy,  % (95% CI) | Usual crude intake,  mean ± SE | Usual adjusted intake,  mean ± SE | Prevalence of inadequacy,  % (95% CI) | Usual crude intake | Usual adjusted intake | Prevalence of inadequacy |
| **3-month midline**^§^ |  |  |  |  |  |  |  |  |  |
| Histidine (mg/d) | 413 ± 8 | 383 ± 7 | 0 (0, 1) | 363 ± 7 | 332 ± 6 | 1 (0, 2) | <0.0001 | <0.0001 | 0.42 |
| Isoleucine (mg/d) | 782 ± 14 | 737 ± 13 | 0 (0, 1) | 666 ± 12 | 621 ± 11 | 0 (0, 1) | <0.0001 | <0.0001 | 0.85 |
| Leucine (mg/d) | 1653 ± 27 | 1527 ± 25 | 0 (0, 1) | 1483 ± 23 | 1355 ± 20 | 0 (0, 1) | <0.0001 | <0.0001 | 0.98 |
| Lysine (mg/d) | 957 ± 21 | 911 ± 20 | 1 (-1, 3) | 785 ± 17 | 740 ± 16 | 14 (8, 21) | <0.0001 | <0.0001 | 0.0002 |
| Sulphur amino acids (mg/d)^¶^ | 656 ± 13 | 611 ± 12 | 0 (0, 1) | 522 ± 9 | 479 ± 8 | 1 (0, 1) | <0.0001 | <0.0001 | 0.74 |
| Aromatic amino acids (mg/d)^¶^ | 1493 ± 27 | 1396 ± 25 | 0 (0, 1) | 1282 ± 22 | 1185 ± 19 | 0 (0, 1) | <0.0001 | <0.0001 | 0.97 |
| Threonine (mg/d) | 677 ± 13 | 637 ± 12 | 0 (0, 1) | 570 ± 10 | 531 ± 9 | 1 (0, 2) | <0.0001 | <0.0001 | 0.52 |
| Tryptophan (mg/d) | 211 ± 4 | 201 ± 4 | 0 (0, 1) | 175 ± 3 | 165 ± 3 | 1 (0, 2) | <0.0001 | <0.0001 | 0.51 |
| Valine (mg/d) | 911 ± 17 | 853 ± 16 | 0 (0, 1) | 761 ± 13 | 703 ± 12 | 1 (0, 3) | <0.0001 | <0.0001 | 0.35 |
| **6-month follow-up**^§^ |  |  |  |  |  |  |  |  |  |
| Histidine (mg/d) | 469 ± 8 | 431 ± 7 | 0 (0, 0) | 422 ± 8 | 384 ± 7 | 0 (0, 0) | <0.0001 | <0.0001 | 0.70 |
| Isoleucine (mg/d) | 853 ± 13 | 797 ± 12 | 0 (0, 0) | 747 ± 12 | 692 ± 11 | 0 (0, 0) | <0.0001 | <0.0001 | 1 |
| Leucine (mg/d) | 1825 ± 27 | 1673 ± 24 | 0 (0, 0) | 1662 ± 25 | 1512 ± 22 | 0 (0, 0) | <0.0001 | <0.0001 | 1 |
| Lysine (mg/d) | 1086 ± 20 | 1026 ± 19 | 0 (0, 0) | 920 ± 20 | 860 ± 18 | 1 (0, 2) | <0.0001 | <0.0001 | 0.12 |
| Sulphur amino acids (mg/d)^¶^ | 720 ± 12 | 666 ± 10 | 0 (0, 0) | 595 ± 11 | 545 ± 9 | 0 (0, 0) | <0.0001 | <0.0001 | 0.74 |
| Aromatic amino acids (mg/d)^¶^ | 1617 ± 25 | 1500 ± 22 | 0 (0, 0) | 1434 ± 21 | 1317 ± 19 | 0 (0, 0) | <0.0001 | <0.0001 | 1 |
| Threonine (mg/d) | 747 ± 12 | 697 ± 11 | 0 (0, 0) | 649 ± 10 | 600 ± 9 | 0 (0, 0) | <0.0001 | <0.0001 | 0.82 |
| Tryptophan (mg/d) | 223 ± 3 | 211 ± 3 | 0 (0, 0) | 192 ± 3 | 180 ± 3 | 0 (0, 0) | <0.0001 | <0.0001 | 0.71 |
| Valine (mg/d) | 998 ± 15 | 927 ± 14 | 0 (0, 0) | 865 ± 14 | 794 ± 12 | 0 (0, 0) | <0.0001 | <0.0001 | 0.76 |

^†^Digestibility-corrected amino acid intakes were calculated by multiplying the amino acid content of each food consumed by its fecal protein digestibility factor, then summing available amino acids from all foods reported in the dietary recall, prior to applying the National Cancer Institute method for estimating mean usual nutrient intakes. Prevalence of inadequacy was estimated based on digestibility-adjusted usual intake and amino acid requirements from the WHO (WHO, FAO, & UNU, 2007).

^‡^Unequal variances t-test for difference between groups using bootstrap standard errors (n=200 bootstrap samples)

^§^Sample size at 3-month midline included n=291 children in the egg group and n=306 children in the control group. Sample size at 6-month endline included n=290 children in the egg group and n=305 children in the control group. Participants were 9 to 12 months old at midline and 12 to 15 months old at endline.

^¶^Sulphur amino acids: methionine and cystine. Aromatic amino acids: phenylalanine and tyrosine.
